# Assessment of the Impact of the Novel "No Sugar Day" Initiative Based on Staff and Student Feedback: A Mixed-Methods Study

**DOI:** 10.7759/cureus.88339

**Published:** 2025-07-19

**Authors:** Ashwin M Jawdekar, Anam Shaikh, Anusree Basu, Shivani Dhonde, Shreyas Neelkanthan, Tejal L Beldar

**Affiliations:** 1 Department of Pediatric and Preventive Dentistry, Bharati Vidyapeeth (Deemed to be University) Dental College and Hospital, Navi Mumbai, IND

**Keywords:** common risk factor approach, noncommunicable diseases, oral health promotion, public health, sugar advocacy, sugar-sweetened beverages

## Abstract

Background

National No Sugar Day emerged from the FDI World Dental Federation and the Indian Dental Association’s Mumbai Declaration on Sugary Drinks and Healthy Foods (June 2022). It was first observed on November 1, 2022, at Bharati Vidyapeeth Dental College, Navi Mumbai. In 2023, the observance expanded to the entire Bharati Vidyapeeth campus, involving students and staff from over 10 institutes, led by the Department of Pediatric and Preventive Dentistry. Activities included recorded and live lectures, a campus march, a “Pledge-Practice-Promote” campaign, a dedicated jingle, and the distribution of printed commemorative mugs. This study aimed to assess the impact of these activities.

Methodology

A retrospective, observational mixed-methods study was conducted to evaluate the perception and impact of the “No Sugar Day” campaign among students and staff at Bharati Vidyapeeth, Navi Mumbai (BVPNM), following ethical approval. The campaign was promoted nationally by the Indian Dental Association (IDA) and locally via posters, e-posters, and a promotional video. Participation was voluntary and limited to those present on campus. Data were collected anonymously within 24 hours using a validated, structured Google Forms (Google, Mountain View, CA, US) shared through institutional WhatsApp groups and email. Descriptive statistics were used for quantitative data, while thematic analysis was applied to qualitative responses. Non-campus participants were excluded. A total of 136 valid responses were analyzed.

Results

The initiative showed a positive impact across several areas. Of the 136 participants, 91.9% (125/136) were aware of No Sugar Day, 86% (117/136) observed it personally, and 91.2% (124/136) promoted it to others. Additionally, 98% (133/136) believed that No Sugar Day is effective in raising awareness about sugar-related health risks (p < 0.00001, chi-square test). Thematic analysis highlighted common sentiments of acceptance, appreciation, creativity, and constructive feedback.

Conclusion

The mixed-methods findings suggest an overall positive short-term impact of the campaign. While symbolic, initiatives like No Sugar Day can support behavior change and create healthier environments at home and in workplaces, contributing to noncommunicable disease prevention efforts.

## Introduction

The World Health Organization (WHO) has identified excessive sugar intake as a major shared risk factor contributing to several noncommunicable diseases, including obesity, diabetes, and cardiovascular conditions, along with its well-established link to dental caries. To mitigate these health risks, WHO recommends reducing free sugar intake to less than 10% of total daily energy intake, with a conditional recommendation to reduce it further to below 5% for added health benefits [1]. In June 2022, an advocacy and capacity-building workshop was jointly organized in Mumbai by the FDI World Dental Federation (FDI) and the Indian Dental Association (IDA). During this event, the concept of a National No Sugar Day was proposed by AMJ (an author of this paper) as a key action item under the “Mumbai Declaration on Sugary Drinks and Healthy Foods.” The declaration was formally signed by the Secretary General of Health from the Ministry of Health and Family Welfare (MoHFW), Government of India (GOI), and endorsed by leading national organizations, including the Food Safety and Standards Authority of India (FSSAI), the Public Health Foundation of India (PHFI), the Indian Academy of Pediatrics (IAP), and the Indian Society of Pedodontics and Preventive Dentistry (ISPPD) [2].

Following the adoption of the declaration, the first No Sugar Day was observed on November 1, 2022, at Bharati Vidyapeeth Dental College and Hospital, Navi Mumbai (BVDCHNM). Encouraged by the initial success, the initiative was significantly scaled up in 2023 to include the entire Bharati Vidyapeeth, Navi Mumbai (BVPNM) campus, engaging 11 constituent colleges. Promotional materials and action plans were also shared with dental institutions across India to encourage voluntary participation. The initiative received international recognition when FDI acknowledged No Sugar Day as a global observance on November 1, as noted in its official position paper [3].

This study aimed to assess the impact of the No Sugar Day observance through a retrospective analysis based on participant feedback. The primary objective was to evaluate how the initiative influenced awareness, behavior, and attitudes among staff and students across various BVPNM colleges. The study employed quantitative methods to assess participation and perceptions and qualitative methods to explore personal experiences and suggestions.

Health observance days have proven effective in raising awareness, promoting behavior change, and influencing public health policy. Initiatives such as World No Tobacco Day, World Oral Health Day, and National Oral Hygiene Day have successfully brought attention to major health issues [4,5]. For example, World No Tobacco Day, observed annually on May 31, has increased global awareness about tobacco-related health risks and supported the implementation of tobacco control legislation [6]. Likewise, in India, Oral Hygiene Day and Oral Cancer Day have improved public understanding of oral health and emphasized the importance of prevention and early detection [7]. Given the increasing concern over high sugar consumption, a shared risk factor for caries, obesity, and other noncommunicable diseases, the WHO has urged population-wide sugar reduction [1]. However, awareness about sugar-related health risks remains limited, especially in developing countries. Advocacy efforts like No Sugar Day offer a promising strategy to localize global health recommendations through education and community-driven behavior change. Drawing inspiration from the success of similar health observances, this study aimed to assess the effectiveness of No Sugar Day as a public health awareness initiative.

The specific objectives of this study were to (1) assess the immediate impact of the No Sugar Day initiative among staff and students across the BVPNM campus; (2) quantitatively evaluate participant engagement, awareness, and behavioral intentions related to sugar consumption following the campaign; (3) qualitatively explore participants’ experiences, attitudes, and suggestions regarding the initiative; and (4) generate recommendations to enhance future observances and align them with broader health promotion efforts.

## Materials and methods

Study design

This research employed a retrospective, observational, mixed-methods design, integrating both quantitative and qualitative approaches to systematically evaluate the impact and perception of the "No Sugar Day" campaign. Ethical clearance was obtained from the Biomedical Ethics Committee of Bharati Vidyapeeth (Deemed to be University) Dental College and Hospital. At the national level, the campaign was promoted by the Indian Dental Association (IDA) through social media and print channels. A promotional video, developed specifically by the principal investigator, was circulated among students and staff of various dental colleges across India. Within the Bharati Vidyapeeth, Navi Mumbai (BVPNM) campus, the campaign was further publicized through posters and e-posters displayed in common areas to maximize visibility among the campus community. Necessary administrative permissions were obtained from the campus director and the principals of all 11 constituent colleges on the BVPNM campus to carry out the campaign activities. All students and staff who were present on campus on the day of the observance were eligible to voluntarily participate in the No Sugar Day activities and provide feedback. Non-teaching staff and individuals who were not on the BVPNM campus during the campaign were excluded from the study.

Data collection

A structured questionnaire was developed using Google Forms (Google, Mountain View, CA, US), incorporating both closed-ended (multiple-choice) questions for quantitative assessment and open-ended questions for qualitative insights. The content of the questionnaire underwent expert validation by two faculty members who were not involved in the study. The survey aimed to capture participants’ awareness, level of participation, perceptions, and feedback regarding the "No Sugar Day" campaign. The questionnaire link was shared via institutional WhatsApp groups and email networks. Participation was voluntary and anonymous, and the form remained open for 24 hours to encourage timely responses without the need for repeated reminders. A total of 136 valid responses were collected during this period (N = 136).

Data analysis

Data were collated and analyzed using Microsoft Excel (Microsoft Corp., Redmond, WA, US). Descriptive statistics (frequencies and percentages) were used to report categorical variables such as participant demographics, awareness, participation, and perceptions of the "No Sugar Day" campaign. Inferential statistics, specifically the chi-square test, were used to examine associations between key variables like designation, awareness, and participation. A significance level of p < 0.05 was considered statistically significant. All closed-ended questions were dichotomous (Yes/No), with "Yes" coded as 1 and "No" as 0. This binary coding enabled straightforward descriptive and inferential analyses. Multiple-choice responses, such as identifying recommended target groups, were similarly coded for each option (1 = Yes, 0 = No). Open-ended responses were analyzed qualitatively through manual thematic analysis and were not numerically scored.

Two independent reviewers conducted the thematic analysis to identify emerging patterns and themes. Any discrepancies were resolved through discussion to ensure consistency and analytical rigor. This mixed-methods approach allowed for a more comprehensive understanding of participant feedback. A total of 136 valid responses were received within the 24-hour window and were included in the final analysis.

Table 1 outlines the structured questionnaire used. Additionally, participant comments from the open-ended section were analyzed to gather deeper insights into perceptions and suggestions related to the campaign.

**Table 1:** Questionnaire administered using Google Forms UG: undergraduate, PG: postgraduate.

Q. no.	Questions (variable of interest)	Response category	Type of variable
1	Gender	M, F	Categorical dichotomous
2	Designation	Teaching staff, UG students, PG students	Categorical polytomous
3	College	Bharati, Non-Bharati	Categorical dichotomous
4	Are you aware that November 1 is National No Sugar Day?	Yes, No	Categorical dichotomous
5	Did you observe November 1 as No Sugar Day?	Yes, No	Categorical dichotomous
6	Did you participate in any No Sugar Day activities in the campus?	Yes, No	Categorical dichotomous
7	Attending the No Sugar Day inaugural program	Yes, No	Categorical dichotomous
8	Participating in the Pledge-Practice-Promote campaign	Yes, No	Categorical dichotomous
9	In-campus march	Yes, No	Categorical dichotomous
10	Wearing a badge	Yes, No	Categorical dichotomous
11	Any other activities	Yes, No	Categorical dichotomous
12	Did you promote No Sugar Day to others?	Yes, No	Categorical dichotomous
13	Social media promotion	Yes, No	Categorical dichotomous
14	Word of mouth promotion	Yes, No	Categorical dichotomous
15	Any other promotion	Yes, No	Categorical dichotomous
16	Do you feel that No Sugar Day is useful for sensitizing people for sugar control?	Yes, No	Categorical dichotomous
17	According to you, No Sugar Day can be recommended to/at:	-	-
	Only self	Yes, No	Categorical dichotomous
	Family and friends	Yes, No	Categorical dichotomous
	Home	Yes, No	Categorical dichotomous
	Workplace	Yes, No	Categorical dichotomous

## Results

The questionnaire used in this research was developed using Google Forms to assess participants' demographic information, as well as their awareness, engagement, and opinions regarding the "No Sugar Day" campaign. It consisted of 17 questions, primarily in a Yes/No (dichotomous) format, focusing on key areas aligned with the study’s objectives. A review informed the development of the questionnaire, drawing on relevant health promotion literature and expert consultation. Two faculty members, who were not involved in the study, reviewed the questionnaire for relevance, clarity, and alignment with the study’s goals. Based on their input, minor revisions were made. A pilot test was conducted with 10 participants representative of the target group to evaluate the clarity, readability, and ease of completion. Based on their feedback, slight wording adjustments were introduced to improve clarity. Although the study was descriptive and observational in nature, with mostly simple dichotomous questions, internal consistency and item relevance were addressed through expert validation and pilot testing.

As this was a retrospective, observational, cross-sectional feedback study, a census-based convenience sampling method was employed. All teaching faculty and students present on the BVPNM campus during the "No Sugar Day" initiative were invited to voluntarily participate by providing their feedback through the questionnaire. Given the study’s design, a formal sample size calculation was not applicable. The goal was to gather as wide a range of feedback as possible from the available population, rather than to obtain a statistically powered sample for inferential analysis. A total of 136 responses were received within the 24-hour collection period, which was considered adequate for the descriptive mixed-methods analysis used in this study.

Both descriptive and inferential statistical methods were used to analyze the variables related to the impact of the No Sugar Day initiative. Dichotomous variables were assessed using the chi-square test, and hypothesis testing was carried out using p-values to determine statistical significance. Open-ended responses were analyzed through manual thematic analysis. Two faculty members with prior experience in qualitative research independently reviewed and coded the responses to identify emerging themes. Any differences in interpretation were resolved through discussion and mutual agreement.

**Figure 1:**
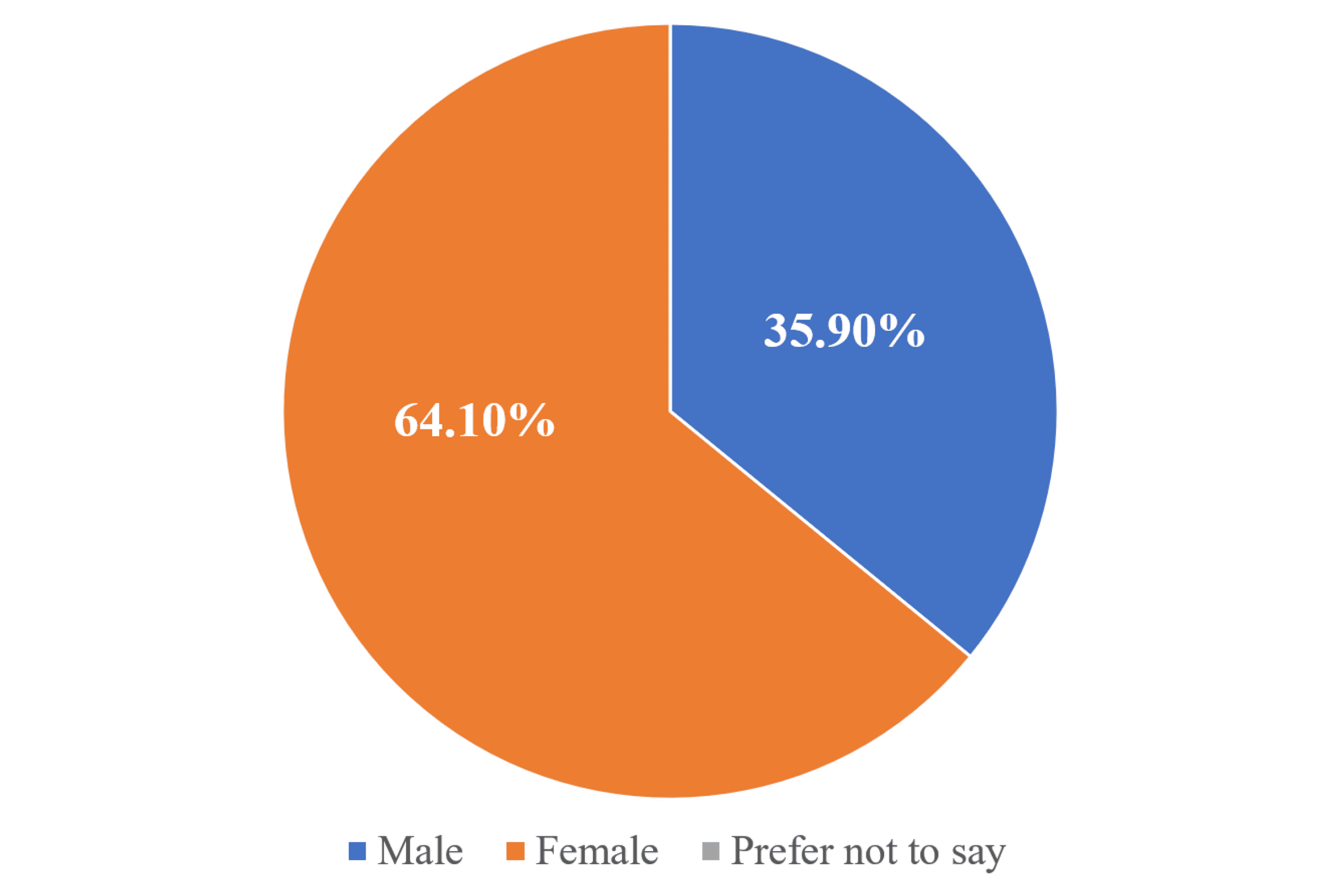
Pie chart showing gender distribution of the participants (N = 136)

This section presents the results of the gender-based analysis, highlighting the observed differences and trends between male and female participants (N = 136). Of the total respondents, 35.9% (49/136) were male and 64.1% (87/136) were female (Figure 1).

**Figure 2:**
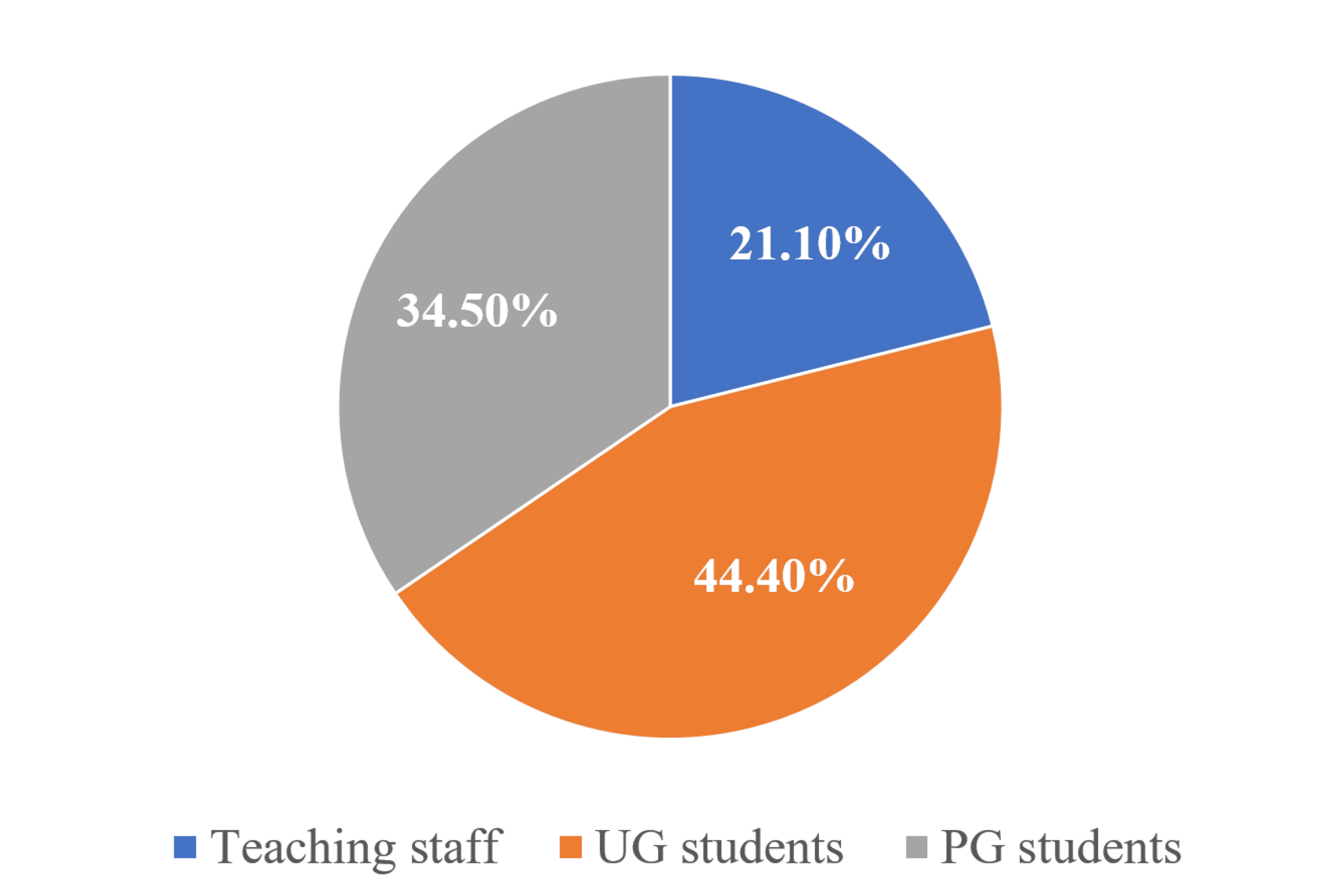
Pie chart showing designation of the participants (N = 136) UG: undergraduate, PG: postgraduate.

The analysis based on designation compared responses among undergraduate students, postgraduate students, and teaching staff. The results showed that 44.4% (60/136) were undergraduate (UG) students, 34.5% (47/136) were postgraduate (PG) students, and 21.1% (29/136) were teaching staff (Figure 2).

**Figure 3:**
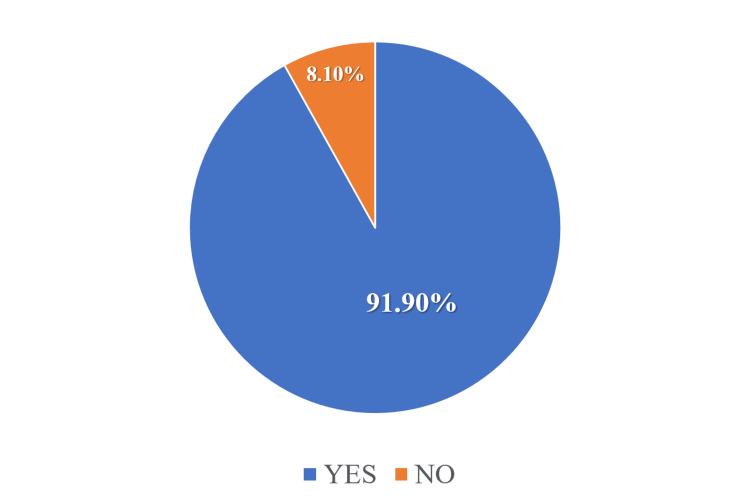
Pie chart showing awareness of the No Sugar Day among participants (N = 136)

The distribution of responses to the question, “Are you aware that November 1 is the National No Sugar Day?” was also analyzed. The findings revealed that 91.9% (125/136) of participants were aware that November 1 is celebrated as No Sugar Day (Figure 3).

**Figure 4:**
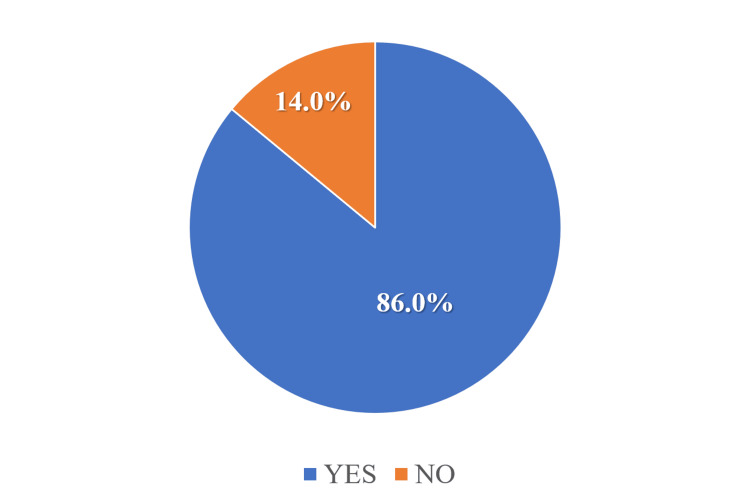
Pie chart showing observation of the No Sugar Day (N = 136)

**Figure 5:**
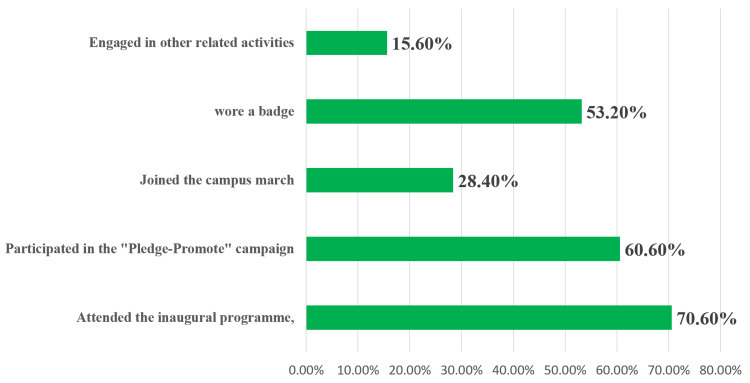
Bar graph showing participation in activities on No Sugar Day (N = 136)

The findings revealed that 86% (117/136) of participants reported actively observing No Sugar Day (Figure 4). Participation in specific activities was as follows: 70.6% (96/136) attended the inaugural program, 60.6% (82/136) took part in the “Pledge-Promote” campaign, 28.4% (39/136) joined the campus march, 53.2% (72/136) wore a badge, and 15.6% (21/136) engaged in other related activities (Figure 5). Additionally, 91.2% (124/136) of respondents indicated that they promoted No Sugar Day to others. Among them, 72.2% (98/136) used social media, 83.8% (114/136) relied on word of mouth, and 7.1% (10/136) used other means of communication.

A significant 97.8% (133/136) of participants believed that No Sugar Day was effective in raising awareness about the health hazards of sugar. Regarding future recommendations, 28.7% (39/136) expressed intent to continue observing the day personally, 92.6% (126/136) recommended it to family and friends, 69.1% (94/136) to household members, and 73.5% (100/136) to their workplace environment. Most of these findings were statistically significant (p < 0.00001), as detailed in Table 2.

**Table 2:** Quantitative assessment of the impact of the No Sugar Day (N = 136) UG: undergraduate, PG: postgraduate.

Sr. no.	Variable of interest	Response category	%	Chi-square value	p-value
1	Gender	M, F	33.1, 66.9	23.12	<0.00001
2.	Designation	Teaching staff, UG, PG	22.1, 44.1, 33.8	10.92	0.004254
3.	College	Bharati, non-Bharati	87.5, 12.5	109.52	<0.00001
4.	Are you aware that November 1 is National No Sugar Day?	Yes, No	91.9, 8.1	141.12	<0.00001
5.	Did you observe November 1 as No Sugar Day?	Yes, No	86, 14	103.68	<0.00001
6.	Did you participate in any No Sugar Day activities in the campus?	Yes, No	70.6, 29.4	35.28	<0.00001
7.	Attending No Sugar Day inaugural program	Yes, No	60.6, 39.4	9.68	0.001863
8.	Participating in the Pledge-Practice-Promote campaign	Yes, No	44, 56	2.88	0.089686
9	Participating in the in-campus march	Yes, No	28.4, 71.6	38.72	<0.00001
10	Wearing a badge	Yes, No	53.2, 46.8	0.72	0.396144
11	Any other activities	Yes, No	15.6, 84.4	92.48	<0.00001
12	Did you promote No Sugar Day to others?	Yes, No	91.2, 8.8	134.48	<0.00001
13	Social media promotion	Yes, No	47.2, 52.8	0.72	0.396144
14	Word of mouth promotion	Yes, No	81.3,18.7	76.88	<0.00001
15	Any other promotion	Yes, No	7.1, 92.9	147.92	<0.00001
16	Do you feel that No Sugar Day is useful for sensitizing people for sugar control?	Yes, No	97.8, 2.2	184.32	<0.00001
17	According to you, No Sugar Day can be recommended to/at:				
	Only self	Yes, No	28.7, 71.3	35.28	<0.00001
	Family and friends	Yes, No	92.6, 7.4	147.92	<0.00001
	Home	Yes, No	69.1, 30.1	28.22	<0.00001
	Workplace	Yes, No	73.5, 26.5	42.32	<0.00001

Qualitative assessment

Several insights emerged from the qualitative assessment of No Sugar Day based on participants’ open-ended responses.

A total of 12 themes were identified, with responses reaching a saturation point due to recurring and similar statements. These themes reflected not only broad acceptance of the initiative but also appreciation for its novelty. Many participants offered constructive suggestions, including expanding the use of various media and promoting behavior change, policy shifts, and environmental adjustments. Respondents also highlighted the perceived health benefits and found the campaign personally and socially motivating. A few suggested integrating No Sugar Day with other health promotion efforts and increasing its visibility through wider public outreach.

In addition to expressing general acceptance and appreciation for the initiative, participants offered creative suggestions for promoting No Sugar Day through various media platforms and settings. They recommended approaches aimed at encouraging behavior change, introducing policy-level interventions, and supporting environmental modifications. Many emphasized the importance of raising awareness about health benefits and exploring healthier alternatives to sugar. Participants also highlighted the value of integrating No Sugar Day with other health promotion activities. Several shared personal experiences and motivations related to reducing sugar intake, reflecting a deeper level of engagement with the campaign.

Table 3 summarizes the key themes along with representative participant responses.

**Table 3:** Qualitative assessment of the impact of the No Sugar Day

Themes	Example quotes
Acceptance	“One day should be there for everyone with no sugar absolutely"
Appreciation	“Sugar is too addictive so let's take baby steps by observing no sugar day”
Creation of more awareness using different media	“Make it a Global No Sugar Day”; “Promote with endorsement of celebrities”
Creation of more awareness in different settings	“Organizing no sugar day camps in schools”; “To go to malls so that mass can be addressed”
Focusing on health benefits	“Little change can bring huge difference in your health”
Behavior change	“Including more fun activities”
Policy change	“It should be written on chocolates and ice cream about harmful effects of sugar, just like it is written on cigarette packs”
Environmental change at workplace/ family	"Promoting alternative of sugar for household use." "Cutting down on tea drinking." Making own fruit juices from fruits rather than drinking artificially sweetened juices from market"
Suggesting an alternative to sugar	“Promoting people to add sugar substitute”
Integrating with other health promotion activities	“Diabetic patients should be made aware of content of sugar and its concentration”
Motivational	“It is not about just a day, sugar should be in control for each and everyday of life”
Own experiences	“Sugar is not good for health. And I had quit (quit) sugar since 1 year. N (And) I observed that I m feeling better then earlier. N even acne are prevented”

## Discussion

Considering the rising prevalence of dental caries, obesity, and diabetes, the WHO released guidelines in 2015 recommending a reduction in the intake of free sugars. Specifically, WHO advised limiting sugar consumption to no more than 10% of total energy intake for both adults and children, citing its clear link to various noncommunicable diseases [1]. In response, on June 17, 2022, the FDI, in collaboration with the IDA, organized the "Whole Mouth Health Advocacy and Capacity Building Workshop on Sugar and Tobacco." This event sparked broad discussions among health leaders and delegates about the long-term harms of sugar consumption. One key outcome was the signing of the "Mumbai Declaration on Sugary Drinks and Healthy Food" on June 24, 2022 [2]. Endorsed by stakeholders such as the MoHFW, the FSSAI, the PHFI, the ISPPD, and others, the declaration proposed seven actionable steps, including the recommendation to observe National No Sugar Day on November 1 every year.

As per the declaration, No Sugar Day was observed on November 1, 2023, at the BVPNM campus. The event aimed to raise awareness and sensitize people about the health risks associated with excessive sugar intake [2]. Past global health campaigns, such as World No Tobacco Day, have demonstrated the power of focused advocacy in promoting awareness and encouraging behavioral change. That initiative successfully drew global attention to the dangers of tobacco and spurred cessation efforts across populations [6]. Similarly, sugar presents a major threat to public health. As one prominent author suggested, sugar may be even more dangerous than gunpowder [8], given its contribution to chronic diseases like dental caries, obesity, and diabetes [9]. Advocacy against sugar requires not only spreading awareness but also fostering environments that support healthy choices. In this context, the symbolic observance of No Sugar Day can serve as a powerful catalyst for change. Our study found high levels of awareness and engagement on campus, with most respondents actively participating and spreading the message through word of mouth. An overwhelming 98% viewed No Sugar Day as an effective awareness tool and expressed willingness to recommend it to others. Qualitative feedback showed strong support, along with constructive suggestions for future iterations, such as involving celebrity endorsements [10], which have already proven effective in boosting public health campaigns. Though initially launched on a limited scale, our findings suggest that No Sugar Day holds strong potential for broader national and even global implementation. India has a strong legacy of leading impactful public health initiatives, and the dental profession, given its direct connection to sugar-related diseases, is well-positioned to lead this campaign.

However, the research is not without limitations. The evaluation relied on real-time feedback, which restricted the ability to assess long-term effectiveness or behavioral change. Sustaining the impact of such campaigns remains a challenge, highlighting the need for continuous and well-structured advocacy efforts. Additional events both before and after No Sugar Day could help reinforce its message. For example, distributing themed mugs served as a reminder of the campaign. Similarly, the "Pledge-Practice-Promote" model introduced during the event could be formally tested in future cycles to assess compliance and long-term impact. Expanding the use of social media with targeted audiovisual content may also enhance outreach. Despite these limitations, No Sugar Day represents a significant milestone in sugar-related health advocacy. This report provides the first systematic evaluation of its impact. The high level of participation and widespread indirect sensitization underscore the campaign’s potential as a scalable and impactful public health intervention.

## Conclusions

The detailed quantitative and qualitative evaluation of participant responses to No Sugar Day indicates its positive and constructive impact in raising awareness and shaping attitudes toward sugar intake for better health. Nearly 92% of respondents were aware of the day, 86% actively observed it, and about 98% believed it was effective in sensitizing people. The high level of participation and engagement suggests that such initiatives can successfully raise awareness about the health risks of excessive sugar consumption, even within academic settings. Though symbolic in nature, No Sugar Day served as a meaningful impetus for sugar advocacy. Beyond increasing knowledge, it encouraged behavioral reflection and motivated participants to make healthier food choices. The campaign also highlighted the strength of inter-institutional and multidisciplinary collaboration in community-based health promotion. Notably, the enthusiasm and willingness of participants to share the message with their families and workplaces indicate the potential for a ripple effect that extends beyond the initial target group. While long-term behavioral impact remains to be studied, No Sugar Day has established a strong foundation. Its continued inclusion in annual health calendars, supported by consistent messaging and partnerships with relevant stakeholders, could contribute significantly to noncommunicable disease prevention. With growing national and international interest, No Sugar Day holds promise as a scalable model for public health advocacy that fosters lasting, health-conscious behavior.

Based on the findings, future initiatives could be strengthened through broader promotion and follow-up assessments to track long-term behavioral change. Expanding the campaign beyond a single campus and including more diverse populations may improve reach and effectiveness. Incorporating structured educational sessions, interactive activities, and online campaigns can reinforce key messages and sustain awareness of the health risks of sugar consumption. Further research using larger, multicenter samples and behavior change models would enhance understanding of the long-term impact and scalability of such health programs.

## Appendices

Authors' note

This research captures the experiences from a single university campus comprising 11 constituent institutes. However, the observance of No Sugar Day extended well beyond this local context. Nationally, around 14 dental colleges participated in the initiative, organizing various awareness and educational activities. The IDA held a national-level competition to recognize impactful No Sugar Day programs, in which BVDCHNM won first prize in the Interdental Collegiate Sweetness Revolution, part of the IDA-FDI project aimed at National Sugar Control Advocacy, supported by the World Dental Development Fund (WDDF). The campaign also received strong support from the ISPPD, which promoted it among its members. Internationally, the campaign gained visibility as the FDI featured it on their website, while countries like Jamaica and Barbados acknowledged and celebrated India's No Sugar Day. In previous years, the Australian Dental Association (ADA) had also recognized the initiative, reflecting its growing international relevance and reach.

**Table 4:** Questionnaire used in this study UG: undergraduate, PG: postgraduate.

Question no.	Variable of Interest	Response category	Variable type
1	Gender	M/F	Categorical dichotomous
2	Designation	Teaching staff, UG students, PG students	Categorical polytomous
3	College	Bharati/non-Bharati	Categorical dichotomous
4	Awareness of the National No Sugar Day	Yes/No	Categorical dichotomous
5	Observance of the No Sugar Day	Yes/No	Categorical dichotomous
6	Participation in campus activities	Yes/No	Categorical dichotomous
7	Attendance at the inaugural program	Yes/No	Categorical dichotomous
8	Participation in the Pledge-Practice-Promote campaign	Yes/No	Categorical dichotomous
9	Participation in an in-campus march	Yes/No	Categorical dichotomous
10	Wearing a badge	Yes/No	Categorical dichotomous
11	Participation in other activities	Yes/No	Categorical dichotomous
12	Promotion of No Sugar Day to others	Yes/No	Categorical dichotomous
13	Social media promotion	Yes/No	Categorical dichotomous
14	Word of mouth promotion	Yes/No	Categorical dichotomous
15	Any other promotion	Yes/No	Categorical dichotomous
16	Perception of usefulness of No Sugar Day	Yes/No	Categorical dichotomous
17	Recommendation of No Sugar Day to:		
	Self	Yes/No	Categorical Dichotomous
	Family and friends	Yes/No	Categorical dichotomous
	Home	Yes/No	Categorical dichotomous
	Workplace	Yes/No	Categorical dichotomous
